# Cellular economy in fission yeast cells continuously cultured with limited nitrogen resources

**DOI:** 10.1038/srep15617

**Published:** 2015-10-21

**Authors:** Yuji Chikashige, Shin'ichi Arakawa, Kenji Leibnitz, Chihiro Tsutsumi, Chie Mori, Hiroko Osakada, Masayuki Murata, Tokuko Haraguchi, Yasushi Hiraoka

**Affiliations:** 1 Advanced ICT Research Institute Kobe, National Institute of Information and Communications Technology, 588-2 Iwaoka, Iwaoka-cho, Nishi-ku, Kobe 651-2492, Japan; 2 Graduate School of Information Science and Technology, Osaka University, 1-5 Yamadaoka, Suita, Osaka 565-0871, Japan; 3 Center for Information and Neural Networks, National Institute of Information and Communications Technology, 1-4 Yamadaoka, Suita, Osaka 565-0871, Japan; 4 Graduate School of Frontier Biosciences, Osaka University, 1-3 Yamadaoka, Suita, Osaka 565-0871, Japan

## Abstract

In ribosome biogenesis, a large fraction of ribosomes is used for producing ribosomal proteins themselves. Here, we applied simulation and experimentation to determine what fraction of ribosomes should be allocated for the synthesis of ribosomal proteins to optimize cellular economy for growth. We define the “r-fraction” as the fraction of mRNA of the ribosomal protein genes out of the total mRNA, and we simulated the effect of the r-fraction on the number of ribosomes. We then empirically measured the amount of protein and RNA in fission yeast cells cultured with high and low nitrogen sources. In the cells cultured with a low nitrogen source, the r-fraction decreased from 0.46 to 0.42 with a 40% reduction of rRNA, but the reduction of the total protein was smaller at 30%. These results indicate that the r-fraction is internally controlled to optimize the efficiency of protein synthesis at a limited cellular cost.

Proliferating cells have a high cost in terms of energy and materials for the biogenesis of ribosomes^1^. Because the ribosome is a tool that synthesizes proteins for cellular proliferation, more ribosomes can produce the necessary proteins more promptly. At the same time, however, ribosomes are also used to produce protein components of the ribosome itself, which competes with the production of proteins necessary for cell proliferation. Thus, it is expected that the fraction of ribosomes used for producing ribosomes is adjusted appropriately to maintain the optimum number of ribosomes for cell proliferation under particular nutritional conditions.

A ribosome consists of four species of rRNA (28S rRNA, 18S rRNA, 5.8S rRNA, and 5S rRNA) and about 80 ribosomal proteins (RPs). Ribosome biogenesis involves three classes of RNA polymerase (Pol I, Pol II, and Pol III). Pol I specifically transcribes RNA components of the ribosome (28S rRNA, 18S rRNA, and 5.8S rRNA); Pol II transcribes mRNA for the protein components of the ribosome (about 80 RPs); and Pol III transcribes another RNA component of the ribosome (5S rRNA). These three classes of RNA polymerase are cooperatively controlled to produce the appropriate number of ribosomes in growing cells^2^. It should be pointed out that Pol II transcribes several thousand genes for non-ribosomal proteins (non-RP) besides RP genes. Thus, we wish to emphasize that transcription of non-RP genes can be influenced when the transcription of the RP genes is controlled for maintenance of the number of ribosomes by Pol II.

In actively growing yeast cells, mRNA of RP genes accounts for about 50% of the total mRNA (ref. 1 and this study). This means that roughly half of the translation initiation reactions on the ribosome are spent on the synthesis of RPs, and the other half on the synthesis of the non-RPs required for cell proliferation. Because a large fraction of ribosomes is involved in the synthesis of ribosomes themselves, allocation of ribosomes for RPs and non-RPs should be appropriately controlled in order to keep the optimal number of ribosomes for cell proliferation. In this study, we carried out simulations and experimental studies of cell proliferation under varying nutritional conditions in the fission yeast *Schizosaccharomyces pombe* to understand the mechanism by which the cell allocates the fraction of ribosomes for ribosome synthesis during cell proliferation.

## Results

### Distribution of mRNA

**Figure 1:**
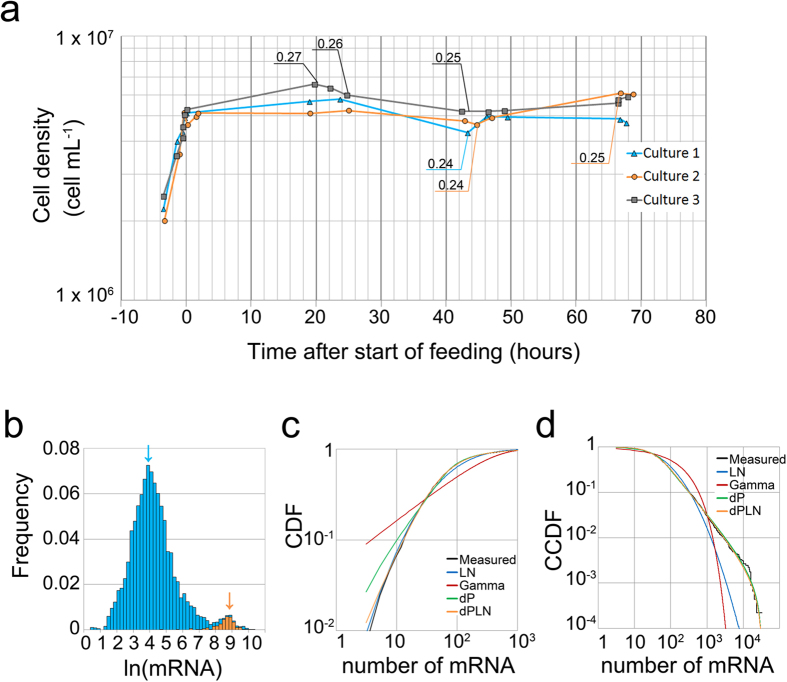
The continuous culture of *Schizosaccharomyces pombe* in the 1 × N EMM2 medium. (**a**) Cell density in three independent continuous cultures in 1 × N-EMM2. The continuous culture started at time = 0. The dilution rate (DR) was set to 0.25 at time = 0 for all cultures. The times when DR was changed are indicated by the DR values in the graphs. (**b**) Histogram of the natural logarithm of the scaled numbers of mRNA in cells continuously cultured in 1 × N-EMM2. The mRNA of ribosomal protein (RP) genes are shown in orange. (**c**,**d**) The distribution of the relative number of mRNA of the 4,536 non-RP genes was well fitted with the dPLN distribution. Cumulative density function (CDF) and complementary CDF (CCDF) are shown in (**c**,**d**), respectively.

The fission yeast *S. pombe* provides an excellent experimental system for the study of cell proliferation in a limited nitrogen resource. Since *S. pombe* cells can grow in a medium including NH_3_ as the sole nitrogen source, it is easy to control the nitrogen concentration in the culture medium. However, the nitrogen concentration cannot be kept constant in a batch culture, which is generally used, because nitrogen in the medium is consumed by cells during cell proliferation. Thus, we cultured cells under a constant nitrogen concentration by using a chemostat, in which a fresh culture medium is continuously provided at a certain rate to replenish the same amount of consumed cell culture (referred to as a continuous culture; see Methods). The results of the continuous culture of the *S. pombe* heterothallic haploid L972 strain in the synthetic medium called EMM2 (including NH_4_Cl 5 g·L^−1^) are shown in Fig. 1a. The maximum dilution rate (DR) of the culture medium, which maintains a constant cell density, was 0.25 per hour on average (SD = 0.0035). The doubling time calculated from the DR was 166 min (SD = 2.3).

Next, we measured the number of mRNA in cells that had been continuously cultured for 72 h in the EMM2 medium by using a DNA microarray. The mRNA of 4678 coding genes corresponding to about 90% of all the protein-coding genes of *S. pombe* was reproducibly detected. The histogram in Fig. 1b shows the distribution of mRNA numbers of the 4678 protein-coding genes based on the DNA microarray experiments. The values on the x-axis are the natural logarithms of the scaled number of each mRNA measured by the DNA microarray (see Methods). A large, almost symmetrical peak (the primary peak, indicated by the blue arrow in Fig. 1b) was observed at the center of the histogram; in addition, a small peak (the secondary peak, indicated by the orange arrow in Fig. 1b) was found in the high-level expression region. This secondary peak was successfully detected because of the improved quantitative accuracy of this study (see Supplementary Notes). Previous studies on a variety of species have reported that the distribution of mRNA is well fitted with heavy-tailed distributions, such as the Pareto distribution or the double Pareto log-normal (dPLN) distribution^3^,^4^,^5^. However, the secondary peak has not been detected before, and therefore has not been taken into account in previous reports. Here, we wish to point out that it is difficult to fit the distribution shown in Fig. 1b with a single distribution function owing to the presence of the secondary peak in the high-level expression region. Thus, we examined the nature of the secondary peak closely. Intriguingly, 129 of the 180 genes included in the secondary peak were RP genes. While *S. pombe* has 142 RP genes (shown in orange in Fig. 1b) coding 79 types of RP components, 90% of the RP genes were included in the secondary peak. By excluding 142 RP genes, the distribution of mRNA in the remaining genes (4536 genes) was well fitted with the dPLN distribution, as shown in Fig. 1c,d. These results indicate that RP genes have a unique profile of gene expression levels.

### Simulation

**Figure 2:**
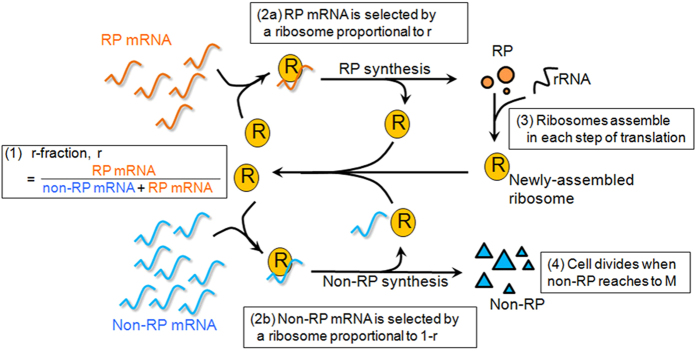
Models for simulation. Illustration of steps (1) to (4) of the simulation algorithm. Ribosomes are partitioned to ribosomal protein (RP) mRNA and non-RP mRNA according to the r-fraction (1). Following the translation of RP mRNA (2a), the newly synthesized RPs are assembled into a new ribosome, which participates in further translation reactions (3). At the same time, a ribosome translates non-RP mRNA to produce non-RPs (2b) that are necessary for cell proliferation (4).

According to the data shown in Fig. 1b, the mRNA of 142 RP genes accounted for 46% (SD = 0.63) of the total mRNA. If the rate of the translational initiation is the same for all mRNA species, 46% of translational initiation reactions are expected to occur for RPs. Following the translation of the RP mRNA, the newly synthesized RPs are assembled into a new ribosome, which participates in further translation reactions. At the same time, a ribosome translates mRNA of many non-RP genes that are necessary for cell proliferation. In proliferating cells, a constant number of ribosomes are maintained and distributed for translation of RPs and non-RPs (Fig. 2). It can be assumed that the fraction of mRNA of RP genes in the total mRNA represents an index approximately showing the fraction of ribosomes used for producing ribosomes. Thus, in the following simulation, we define “r-fraction” as the fraction of mRNA of RP genes out of the total mRNA. To clarify the relationship between the number of ribosomes and the r-fraction in proliferating cells, we designed a simulation based on the steps of the following algorithm (Fig. 2).

1. The total number of mRNA is divided into two groups: mRNA for the RP and non-RP genes, according to the r-fraction. The ratio of mRNA in each group is constant throughout the whole process.
2. Each mRNA molecule has an equal chance of being selected by a ribosome to produce proteins during the process of translation. Therefore, the probability of each protein being produced is proportional to the number of mRNA of each protein gene. The number of mRNA for each gene is provided by the microarray data.
3. The start and end of translation of all ribosomes in the cell is synchronized. This round of translation is defined as one iteration. One ribosome is assembled by one set of 79 types of RP. Thus, the number of ribosomes in the next iteration increases by the fewest proteins of each of the 79 types of RP synthesized at the previous iteration. All ribosomes in the cell participate in translation.
4. A critical protein number M is given to determine when the cell divides. The cell divides when the number of non-RPs newly synthesized exceeds that M value. At the time of cell division, ribosomes are equally distributed to the daughter cells and RPs that have not been assembled into a ribosome are eliminated.

At the start of the simulation, all the RPs have been assembled into a ribosome. The initial number of ribosomes (R_0_) and the critical protein number M are given at the start of each simulation.

**Figure 3:**
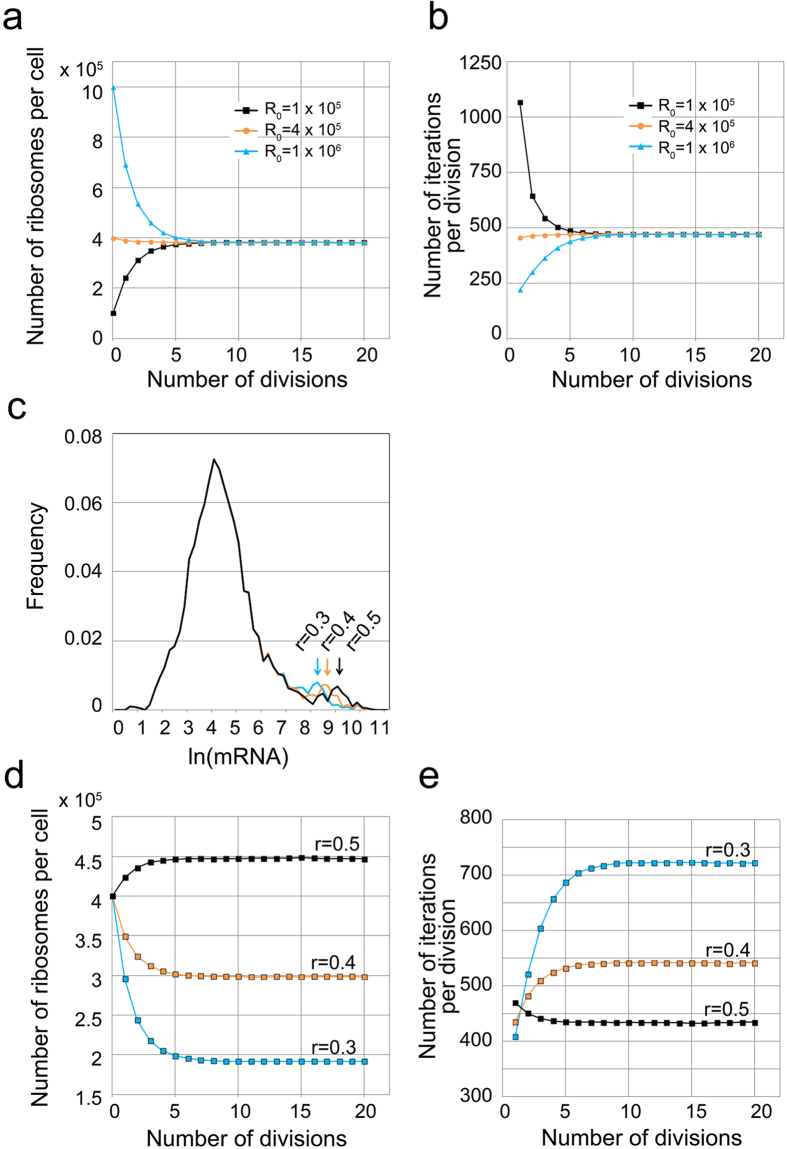
Results of the simulation. (**a**,**b**) Simulation results of the number of ribosomes (**a**) and the number of iterations required for cell division (**b**) as a function of number of divisions. The number of mRNA for each gene experimentally determined in 1 × N-EMM2 (r-fraction of 0.46) was used and the parameters M = 1.4 × 10^8^ and R_0_ = 1 × 10^5^, 4 × 10^5^, or 1 × 10^6^ were given in each simulation. The x-axis indicates the number of divisions, the y-axis indicates the number of ribosomes of each cell just after the cell division in (**a**), and the number of iterations required for division in (**b**). (**c**) Histogram of mRNA where the r-fraction (abbreviated as r) is set to 0.3, 0.4, and 0.5. (**d**,**e**) Simulation of the number of ribosomes and the number of iterations required for cell division for three values of the r-fraction, in which M = 1.4 × 10^8^ and R_0_ = 4 × 10^5^ were given, and the number of mRNA for each gene was the same as in (**a**) except in ribosomal protein (RP) genes.

The results of the simulation are shown in Fig. 3. In this simulation, the critical protein number M was set to 1.4 × 10^8^; the initial number of ribosomes R_0_ was set to 1 × 10^5^, 4 × 10^5^, or 1 × 10^6^. An r-fraction value of 0.46 was used corresponding to the measurements in the cells continuously cultured in EMM2. In Fig. 3a,b, the x-axes indicate the number of divisions, and the y-axes indicate the number of ribosomes in each cell just after cell division and the number of iterations required for division, respectively. As shown in Fig. 3a,b, the number of ribosomes for each cell and the number of iterations at each division approached a constant value irrespective of the initial number of ribosomes (R_0_). Figure 3c shows the mRNA distribution for different values of the r-fraction (abbreviated to r) where r = 0.3, 0.4, and 0.5. This distribution was derived from the r-fraction shown in Fig. 1b by multiplying the number of mRNA of 142 RP genes with a constant value. The position of the secondary peak found in Fig. 1b shifted from left to right as the r-fraction increased. Figure 3d,e show the simulation results for the number of ribosomes and the number of iterations required for cell division, respectively, for three values of the r-fraction (r = 0.3, 0.4, and 0.5), in which M = 1.4 × 10^8^ and R_0_ = 4 × 10^5^ were given and the number of mRNA for each gene was the same as in Fig. 3a except for the RP genes. The number of ribosomes for each cell decreased by 33–36% as the r-fraction decreased by every 0.1 (Fig. 3d). A larger r-fraction caused cells to proliferate with fewer iterations (Fig. 3e). These simulation results evoke the question of whether the number of ribosomes is adjusted in living cells by changing the r-fraction — in other words, by shifting the second peak in the mRNA distribution. To answer this question, we next measured RNA levels in the cells that proliferated in low-nitrogen conditions, expecting that ribosomes would decrease.

### Low-nitrogen culture

Cells of *S. pombe* heterothallic haploid strains stop proliferation when nitrogen in the culture medium is exhausted, and the cell cycle is blocked at the G1 phase^6^. In continuous culture, the cell can proliferate without exhausting nitrogen because the nitrogen concentration is kept constant in the medium while cell growth is maintained. We investigated whether *S. pombe* cells could proliferate in a series of different NH_3_ concentrations by using the EMM2 medium, and found that the cells could proliferate at 100 times lower NH_3_ than in the standard EMM2 medium (we refer to it as 1/100 × N-EMM2 and the standard EMM2 as 1 × N-EMM2 in the following). The NH_3_ concentration at the 1/100 × N-EMM2 was about 0.93 μmol·mL^−1^; the amount of NH_3_ for each cell was about 190 fmol at the cell density of 5 × 10^6^ cell·mL^−1^. On the other hand, the amount of nitrogen included in the cell was estimated at about 170 fmol for a cell volume of 80 fL (see Methods). Thus, 190 fmol of nitrogen is almost equal to the amount of nitrogen included in one cell, and is expected to be consumed in one cell cycle. Based on this estimation, we used 1/100 × N-EMM2 as the minimal concentration of nitrogen that allows *S. pombe* cells to continue proliferation.

**Figure 4:**
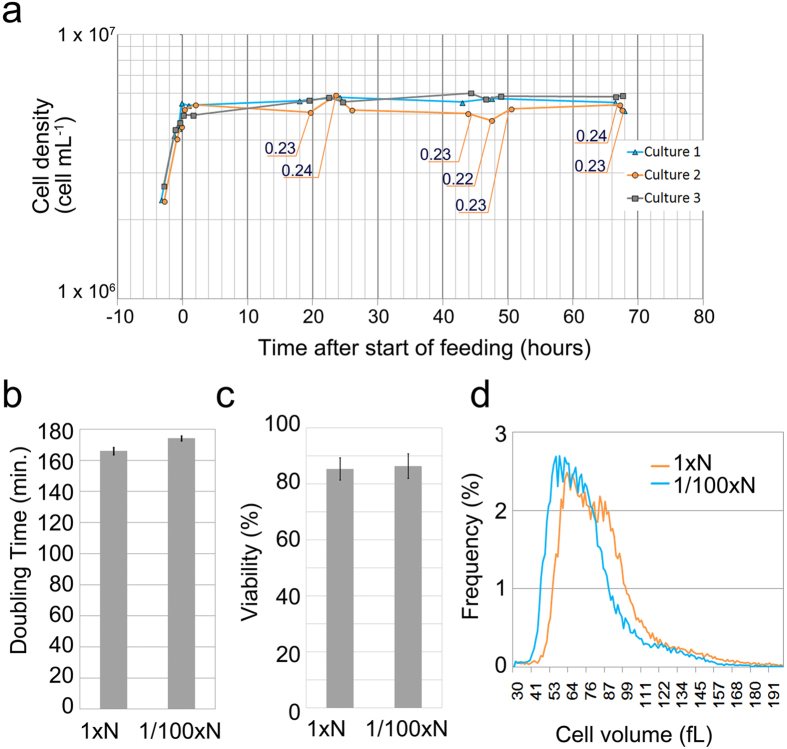
The continuous culture of *Schizosaccharomyces pombe* in the 1/100 × N-EMM2. (**a**) Cell density in three independent continuous cultures in 1/100 × N-EMM2. The continuous culture started at time = 0. The dilution rate (DR) was set to 0.24 at time = 0 for all cultures. The times when the DR was changed are indicated by the DR values in the graphs. There were no DR changes in cultures 1 or 3. (**b**) Doubling times calculated from the DRs for each culture condition. Average values shown are 166 minutes (SD = 2.3) for 1 × N-EMM2 and 174 minutes (SD = 1.6) for 1/100 × N-EMM2 in four independent cultures; the error bars show the standard deviation. (**c**) Cell viability was measured by colony formation efficiency at two time-points in two independent continuous cultures for each culture condition. The error bars show the standard deviation among the four measurements. Averaged values shown are 85.4% (SD = 3.98) for 1 × N-EMM2 and 86.4% (SD = 4.42) for 1/100 × N-EMM2. (d) Histogram of cell volumes measured by using a particle analyzer. Values shown are the average of four independent experiments

The results of continuous cultures in 1/100 × N-EMM2 are shown in Fig. 4a. The maximum DR of the culture medium was 0.24 (SD = 0.0022), which was slightly lower than that in 1 × N-EMM2. The doubling time calculated from the DR was approximately 174 minutes (SD = 1.6), 5% longer than that in 1 × N-EMM2 (Fig. 4b). Comparing the efficiency of colony formation on the YES plate during continuous culture in 1 × N-EMM2 and 1/100 × N-EMM2, there were no differences in the viability of the cells between these two nitrogen concentrations (Fig. 4c). The average cell volume was 75.2 fL (SD = 0.92), 12% smaller than that in 1 × N-EMM2 (85.5 fL; SD = 0.50) (Fig. 4d).

### Gene expression in low-nitrogen culture

**Figure 5:**
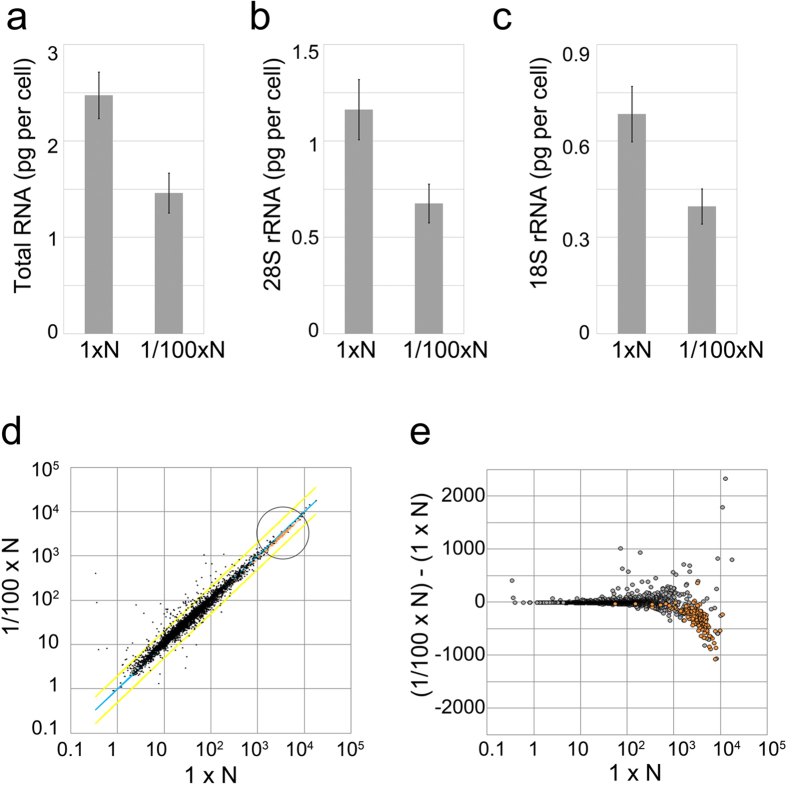
Measurements of RNA in the continuous cultures. (**a**–**c**) Amount of total RNA (**a**), 28S rRNA (**b**), and 18S rRNA (**c**) per cell cultured in 1 × N-EMM2 and 1/100 × N-EMM2. Values shown are the average of five independent experiments for 1 × N-EMM2 and four independent experiments for 1/100 × N-EMM2: 2.47 pg per cell (SD = 0.24) and 1.46 pg per cell (SD = 0.21) (**a**), 1.16 pg per cell (SD = 0.16) and 0.68 pg per cell (SD = 0.10) (**b**), 0.68 pg per cell (SD = 0.086) and 0.40 pg per cell (SD = 0.055) (**c**) for 1 × N-EMM2 and 1/100 × N-EMM2, respectively. The error bars show the standard deviation among experiments. (**d**) Scatter plot of the mRNA numbers in 1 × N-EMM2- and 1/100 × N-EMM2-cultured cells in double logarithmic scales. Each dot corresponds to one gene. The blue diagonal line shows the relative mRNA numbers that are equal in the 1/100 × N-EMM2-cultured cells and in the 1 × N-EMM2-cultured cells. The two yellow diagonal lines indicate the range in which the relative mRNA numbers in the 1/100 × N-EMM2-cultured cells are within twice the numbers of the 1 × N-EMM2-cultured cells. Orange dots correspond to ribosomal protein (RP) genes. The circle highlights the area of high expression genes. (**e**) Differences in the relative number of mRNA between the 1 × N-EMM2- and the 1/100 × N-EMM2-cultured cells (y-axis) are plotted against the relative number of mRNA in the 1 × N-EMM2-cultured cells (x-axis, logarithmic scale). Orange dots correspond to RP genes.

In our measurements, the amount of total RNA per cell was 2.47 pg (SD = 0.24) and 1.46 pg (SD = 0.21) in the 1 × N-EMM2 and 1/100 × N-EMM2 cultures, respectively. We also measured the ratio of 18S and 28S rRNA included in the total RNA by using a BIO-analyzer (Agilent). In addition to the total amount of RNA, the amounts of both rRNA species in the 1/100 × N-EMM2-cultured cells decreased by about 40% compared with those in the 1 × N-EMM2-cultured cells (Fig. 5a–c). Thus, it can be assumed that the number of ribosomes decreases by about 40% in the 1/100 × N-EMM2 culture. We next measured the level of gene expression by DNA microarray analysis using RNA isolated from the 1/100 × N-EMM2-cultured cells. To compare relative values of gene expression, we scaled the microarray data by normalizing the total number of mRNA per cell in the 1 × N-EMM2 and 1/100 × N-EMM2 cultures to be equal in number (Fig. 5d,e; see Methods for details). The ratio of the mRNA expression profile between the 1 × N-EMM2- and 1/100 × N-EMM2-cultured cells was 0.5–2 for 4602 out of 4722 genes (97%), as indicated by the yellow lines in Fig. 5d. This suggests that the absolute number of mRNA for most of the genes decreased proportionally when the total amount of RNA was reduced by 40% in the 1/100N-EMM2 culture. Our results for the proportional decrease of mRNA levels suggest that responses to nitrogen starvation did not occur in the 1/100 × N-EMM2 culture because mRNA expression would have been induced by more than four times in over 1000 genes if nitrogen starvation had occurred in *S. pombe*^7^.

Among the 4722 genes detected, 59 were reproducibly found in four independent experiments that increased their relative mRNA levels by more than twice in the 1/100 × N-EMM2 culture compared with the 1 × N-EMM2 culture (Table 1). In these genes, the absolute mRNA levels increased, or decreased less, compared with the many other genes. In a previous report^7^, the genes induced by nitrogen starvation are classified into four groups according to the stages at which they are expressed: (1) the starvation/pheromone induction, (2) early meiosis, (3) middle meiosis, and (4) late meiosis. Thirty-eight genes out of 59 in Table 1 were included in these four groups: 35 in the starvation/pheromone induction, 2 in middle meiosis, and 1 in late meiosis. Genes related to amino acid transport and amino acid metabolism were included in the list of 59 genes. Interestingly, the *ste11* gene, one of the central regulators that induce meiosis upon nitrogen starvation^8^, did not increase in the 1/100 × N-EMM2-cultured cells. It follows that the cells detected the shortage of nitrogen sources and continued proliferation in the 1/100 × N-EMM2 culture condition without arresting the cell cycle, unlike in the response to nitrogen starvation.

### Distribution of mRNA in low-nitrogen culture

**Figure 6:**
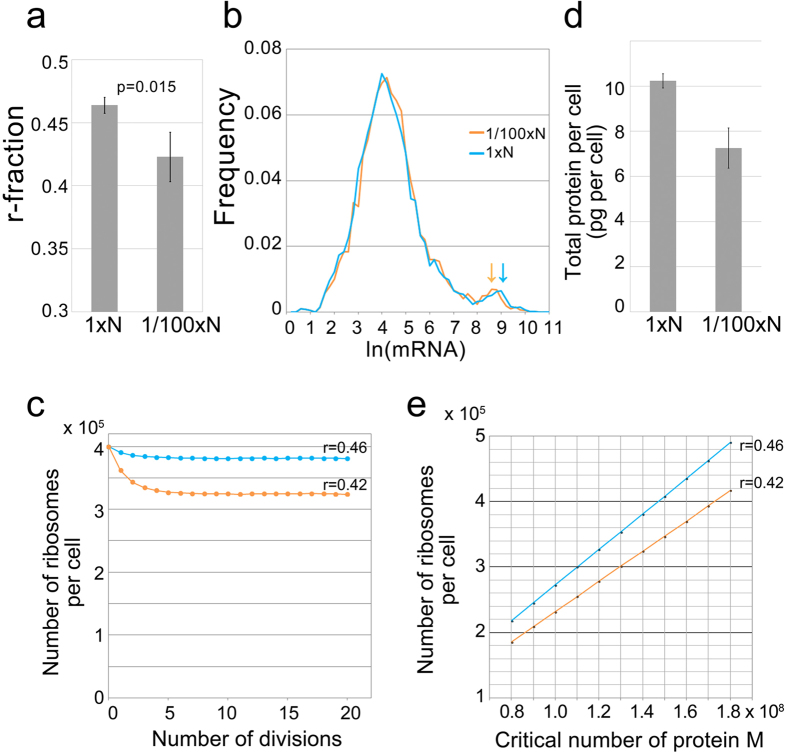
Comparison of the simulation and the empirical measurements. (**a**) r-fraction in cells continuously cultured in 1 × N-EMM2 and 1/100 × N-EMM2. Values shown are averages of four independent experiments: 0.46 (SD = 0.0063) for 1 × N-EMM2 and 0.42 (SD = 0.020) for 1/100 × N-EMM2. The error bars show the standard deviation among experiments. (**b**) Histogram of mRNA in cells continuously cultured in 1 × N-EMM2 and 1/100 × N-EMM2. The arrows indicate the secondary peak. (**c**) Simulation of the number of ribosomes at the critical protein number M = 1.4 × 10^8^ combined with two values of the mRNA number in the 1 × N-EMM2-cultured cells (r-fraction = 0.46) and that in the 1/100 × N-EMM2-cultured cells (r-fraction = 0.42). (**d**) Total amount of proteins per cell cultured in 1 × N-EMM2 and 1/100 × N-EMM2. Values shown are averages of three independent experiments: 10.2 pg per cell (SD = 0.32) for 1 × N-EMM2 and 7.25 pg per cell (SD = 0.89) for 1/100 × N-EMM2. The error bars show the standard deviation among experiments. (**e**) Number of ribosomes are plotted against the critical protein number M in the simulation. The numbers of ribosomes shown are those after 20 cell divisions.

The relative number of mRNA in most of the genes in the 1/100 × N-EMM2 culture changed by less than twice compared with the 1 × N-EMM2 culture, as mentioned above (dots scattered above and below the yellow diagonal line in Fig. 5d). In the high-expression region (marked by the circle in Fig. 5d), many genes exhibited a reduction in the relative number of mRNA in the 1/100 × N-EMM2 culture compared with that in the 1 × N-EMM2 culture (dots accumulated below the blue diagonal line). This is more clearly seen in Fig. 5e, in which the difference in the relative number of mRNA between 1 × N-EMM2- and 1/100 × N-EMM2-cultured cells (y-axis) is plotted against the relative number of mRNA in the 1 × N-EMM2-cultured cells (x-axis). Many RP genes (plotted in orange) are included in the group of genes whose relative number of mRNA decreased in the 1/100 × N-EMM2 culture in the high-expression region. Reflecting these results, the r-fraction decreased to 0.42 (SD = 0.02) in the 1/100 × N-EMM2 culture from 0.46 in the 1 × N-EMM2 culture (Fig. 6a). In the histogram showing the distribution of mRNA in the 1/100 × N-EMM2-cultured cells, the secondary peak shifted slightly to the left compared with that in the 1 × N-EMM2-cultured cells (Fig. 6b). We then simulated the number of ribosomes in the 1/100 × N-EMM2-cultured cells under the same conditions as in Fig. 3a except that we used the data for the mRNA number in the 1/100 × N-EMM2 culture (r = 0.42). The result of this simulation is shown in Fig. 6c. The number of simulated ribosomes in the 1/100 × N-EMM2-cultured cells was approximately 324,000, i.e., a decrease of only about 15% from that in the 1 × N-EMM2 culture. The reduction of the r-fraction from 0.46 to 0.42 can only partly explain the decrease of ribosomes, as our experiments estimated the number of ribosomes to be reduced by about 40% in the 1/100 × N-EMM2 culture compared with that in the 1 × N-EMM2 culture based on measurements of total RNA per cell.

### Critical protein number in low-nitrogen culture

There was a significant difference in the reduction in the number of ribosomes in the 1/100 × N-EMM2-cultured cells between the simulated value and the value estimated by RNA measurements. In this simulation, the same value of the critical protein number M was used for simulating the 1 × N-EMM2 and 1/100 × N-EMM2 conditions. However, the value of M, which represents the number of non-RPs required for cell division, may vary according to the nutritional conditions. Thus, the discrepancy between simulations and experiments might arise from the application of the same fixed value of M to high- and low-nutrient conditions, and different M values may need to be applied for different conditions.

To consider this possibility, we measured the amounts of total protein per cell in each condition, and found that the total amount of protein per cell decreased by about 30% in the 1/100 × N-EMM2-cultured cells (7.25 pg per cell; SD = 0.89) compared with the 1 × N-EMM2-cultured cells (10.2 pg per cell; SD = 0.32) (Fig. 6d). A reduction of the amount of total protein was observed in the 1/100 × N-EMM2-cultured cells, suggesting that the critical protein number M should be appropriately reduced in the simulation for low nitrogen sources.

Thus, we conducted a simulation by adopting the following conditions: values for the critical protein number M ranged from 0.8 × 10^8^ to 1.8 × 10^8^ combined with two values of mRNA number in the 1 × N-EMM2-cultured cells (r = 0.46) and in the 1/100 × N-EMM2-cultured cells (r = 0.42). The results of the simulation are shown in Fig. 6e. When the r-fraction was fixed, the number of ribosomes seemed to be proportional to the M value, which means that a 30% lower M value led to a 30% reduction in ribosomes (i.e., 70% ribosome production). On the other hand, reduction of the r-fraction from 0.46 to 0.42 reduced the number of ribosomes by 15% for each M value (i.e., 85% ribosome production). When the 30% reduction in M value was combined with the r-fraction reduction from 0.46 to 0.42, the decrease in the number of ribosomes was 40% (i.e., 70% × 85% ≈ 60% ribosome production). In the experiments, when the ribosomes were reduced by 40%, as estimated by the amount of rRNA, the total protein was reduced at the lower level of 30% (Fig. 6d). This difference can be explained by the adaptation of the r-fraction from 0.46 to 0.42, as predicted by simulations. The reduction of the r-fraction caused the reduction of ribosome synthesis and thus resulted in the allocation of more ribosomes for non-RPs. These results indicate that the r-fraction is internally controlled to optimize the efficiency of protein synthesis by reacting to nutritional environments.

## Discussion

Since ribosomes are necessary for the synthesis of essential cell proteins, including the proteins of the ribosomes themselves, there is a trade-off between RPs and non-RPs in the use of ribosomes for synthesis. This trade-off is more critical for cell survival when resources are limited. In this report, we attempted to determine how many of the ribosomes are allocated to produce ribosomes when *S. pombe* cells were cultured with limited nitrogen sources. Such experiments have not been possible with a batch culture, but they have become possible by continuous culture.

To evaluate the fraction of ribosomes used to produce ribosomes, we defined the r-fraction as the fraction of the RP mRNA in the total mRNA. Our experimental measurements suggest that RP genes are controlled by a separate mechanism distinguished from non-RP genes, forming the characteristic secondary peak in the mRNA distribution; a change in the r-fraction causes a shift of the secondary peak (Fig. 3c).

Simulations indicate that when the r-fraction is given at a fixed value of M, the number of ribosomes per cell and the number of iterations for cell division reaches a constant value (Fig. 3a,b). Simulation also predicts the consequences of changing the r-fraction in growing cells (Fig. 3d,e): the number of iterations decreases and the number of ribosomes increases as the r-fraction increases. When the r-fraction is constant, the critical number of proteins is proportional to the number of ribosomes. Therefore, increasing the number of ribosomes without changing the r-fraction under unlimited resources can increase the critical number of proteins. However, when resources are limited, the number of ribosomes decreases, as shown empirically. Simulation shows that a reduced number of ribosomes can produce an increased critical number of proteins by lowering the r-fraction (Fig. 6e).

This is consistent with the experimental data; in the cells cultured in low nitrogen, the amount of total RNA and the amount of total rRNA both decreased by 40%, but the total amount of protein decreased by only 30% and the r-fraction decreased to 0.42 compared with 0.46 in the high-nitrogen culture. If the r-fraction did not change, the total protein decreased to the same extent as the decrease in rRNA. However, by reducing the r-fraction from 0.46 to 0.42, the cells were able to keep the total protein at a lower level of reduction (30%) while the rRNA decreased by 40%. Thus, a small reduction of the r-fraction can allocate limited resources to produce essential proteins more efficiently.

It is generally thought that the cell maintains the adequate number of ribosomes by controlling transcription of rRNA using Pol I in response to the environment^9^. In addition, it is also thought that RP mRNA transcription by Pol II and 5S rRNA transcription by Pol III are controlled cooperatively^2^. The cell employs the mechanism in which transcription of 28S/18S/5.8S rRNA by Pol I always changes in correlation with transcription of RP mRNA by Pol II. However, the level of non-RP mRNA has not been considered in the context of ribosome synthesis. In this report, we illustrated the influence of the level of non-RP mRNA on ribosome synthesis by introducing the r-fraction. As discussed above, if the r-fraction is unchanged, RP and non-RP decrease proportionally when the number of ribosomes decreases; in contrast, by reducing the r-fraction, the level of non-RP can be maintained while RP is reduced. Indeed, our experiments showed that the amount of rRNA and the r-fraction both decreased in the low-nitrogen culture. Thus, we propose that the cell has internal mechanisms to adapt to the nutritional environment not only by changing the number of ribosomes but also by modulating allocation of ribosomes for ribosome synthesis (i.e., r-fraction) to maintain the homeostasis of protein synthesis.

## Methods

### Continuous culture of *S. pombe* cells

The *S. pombe* 972 *h*^*−*^ strain was used in all experiments. The EMM2 medium^10^ was used for the 1 × N-EMM2 culture, and the EMM2 medium, in which the concentration of NH_4_Cl was changed to 0.05 g·L^–1^, was used for the 1/100 × N-EMM2 culture. Continuous culture was carried out with a chemostat and feed controller BMJ-01NC (ABLE-Biott) at 30 °C and 1.5 vvm (air volume per culture volume per minute) aeration, and stirred with a blade turbine at 600 rpm. The pH of the medium was 5.8. For experiments with the 1 × N-EMM2 medium, the *S. pombe* cells in the batch culture in EMM2 were transferred at mid log phase to the vessel after washing once with the EMM2 medium to produce 2 × 10^6^ cells·mL^−1^ in 600 mL EMM2. After culturing for some hours to grow at 5 × 10^6^ cells·mL^−1^ without feeding, the continuous culture started at a specific DR. During the continuous culture, the cell concentration was kept constant by removing the cell culture at a certain DR and continuously providing a fresh culture medium at the same rate as cells continued proliferating. When the DR was 0.25 per hour, 2.5 mL culture was continuously replaced for each minute [2.5 mL = 600 mL (culture volume) × 0.25 (DR)/60 (minutes)]. We set the DR at the maximum possible rate that did not wash out the cells. The DR was occasionally adjusted to keep the cell density at around 5 × 10^6^ cells·mL^−1^. The DRs during 72 h culture were averaged. Doubling time (DT) can be calculated from the DR with DT = ln(2)/DR, as described^11^. For the experiment with the 1/100 × N-EMM2 medium, the pre-batch culture was carried out in EMM2 with 16 times diluted NH_4_Cl and washed with the EMM2 with 1/100 × N. Density and viability of cells during culture were checked with a small volume (e.g., 2.5 mL) taken from the culture: when the DR was 0.25 per hour, the volume of culture taken was 2.5 mL corresponding to a one-minute flow. After these samplings, the feed-out pump was stopped for one minute to recover the culture volume. Cell density and cell volume were measured with the particle analyzer CDA-1000 (Sysmex). Cell viability was checked as follows: 300–500 cells for each sample taken at 48 h and 72 h were dissected on YES plates with the dissection microscope MSM (Singer Instruments). After 3 days of incubation at 30 °C, the colonies were counted.

### Estimation of amount of nitrogen per cell

The amount of nitrogen in a budding yeast cell is reported to account for 8% of its dry weight^12^ and water accounts for about 60% of the weight of a wet yeast cell^13^. According to the budding yeast values, we estimated the amount of nitrogen of fission yeast to be about 3% in weight of a living cell. The density of a living cell was assumed to be 1 pg·fL^−1^.

### Measurements of total protein and RNA

For calculating the average amount of total protein and RNA for a cell, the total protein and RNA should be isolated from a known number of cells. After 72 h of continuous culture, cells were harvested by filtration. Then, the wet pellet of cells was divided into three parts and the weight of each part was measured. One of the three parts was suspended in a fixed volume of water and the cell number of the cell suspension was counted with the particle analyzer CDA-1000 (Sysmex) to give the number of cells in the wet pellet per weight. Total protein and RNA were isolated from the remaining two parts of pellet.

Total protein was isolated and measured as follows: the cell pellet containing approximately 3 × 10^7^ cells was suspended in 100 μL of the lysis buffer (2% sodium dodecyl sulfate (SDS); 10% glycerol; 60 mM Tris-HCl, pH 6.6; 8 M urea) and boiled at 100 °C for 5 min, then cooled on ice. Cells were disrupted with 0.5 mm glass beads using the Multi-Beads Shocker (Yasui Kikai) at 2,700 rpm for 1 min (repeated four times). The resulting cell extracts were centrifuged at 100,000 *g* for 10 min. After recording the volume of the supernatant, its protein concentration was measured using a Bio-Rad DC Protein Assay Kit (Bio-Rad).

Total RNA was isolated and measured as follows. The cell pellet was frozen with liquid nitrogen. The frozen cell pellet was mixed with 500 μl of TES (10 mM Tris pH 7.5, 10 mM ethylenediaminetetraacetic (EDTA) pH 8, 0.5% SDS) and 500 μl of acidic phenol-chloroform on ice. The sample was incubated at 65 °C for 1 hour, and then placed on ice for 1 min. The sample was transferred to a phase lock tube (MaXtractTM HighDensity 2 ml Qiagen) and was centrifuged for 5 min at 15100 *g* at 4 °C. The water phase of the sample (500 μl) was mixed with 500 μl of acidic phenol-chloroform in a new phase lock tube. The sample was centrifuged for 5 min at 15100 *g* at 4 °C. The water phase of the sample (500 μl) was mixed with 500 μl of chloroform in a new phase lock tube. The sample was centrifuged for 5 min at 15100 *g* at 4 °C. Total RNA was purified from the water phase by ethanol precipitation. After resolving the RNA pellet in a fixed volume of water, the concentration of the isolated RNA was measured with a ND-1000 spectrophotometer (NanoDrop Technology). A small portion of the isolated RNA was also analyzed with a BIO-analyzer 2100 (Agilent) to check its purity and RNA composition.

### DNA microarray experiments

Cyanine-3 (Cy3)-labeled cRNA was prepared from 200 ng RNA using the LowInput QuickAmp Labeling Kit, one color including Cy3-CTP (Agilent Technologies) according to the manufacturer’s instructions, followed by RNeasy mini column purification (QIAGEN, Valencia, CA). Hybridization was carried out using the Gene Expression Hybridization Kit (Agilent Technologies) according to the manufacturer’s instructions except for the amount of Cy3-labeled cRNA (37.5 ng was used for each hybridization). After hybridization, microarrays were washed for 1 minute at room temperature with GE Wash Buffer 1 (Agilent) and for 1 minute with GE Wash buffer 2 (Agilent) at 37 °C, then immediately dried. Slides were scanned immediately after washing on the Agilent DNA Microarray Scanner (G2505C) using one color scan setting for 8 × 60 K array slides (scan area: 61 × 21.6 mm; scan resolution 3 μm; dye channel set to green; and green PMT set to 100%). The scanned images were analyzed using Feature Extraction Software (ver. 10.7.3.1, Agilent) using default parameters (protocol GE1-107_Sep09 and Grid: 038274_D_F_20120123) to obtain background-subtracted and spatially detrended processed signal intensities.

### Microarray data processing

The 6,767 probes (including 6711 *S. pombe* sequences and 56 control probes) were spotted multiply to fill 62,976 spots in each array. The median of the *gProcessedsignals* and an average of the *gIsWellAboveBG* (*gProcessedsignals* and *gIsWellAboveBG* are defined by the software Feature Extraction) of the spots for each probe were calculated to make a data set for 6,767 probes. When the average of *gIsWellAboveBG* of the spots for each probe was higher than 0.55, the probe signal was considered to be above the detection limit. The median was normalized by the 75 percentile method (the value at 75% was set to 2,500) to obtain values for the 6,767 probes. The measured values for these spots were assigned to the 6,657 *S. pombe* genes currently annotated in a public database. The values of probes that represent duplicated genes were divided by the number of duplicated genes to give corrected values to the genes. The values of probes that were cross-hybridized with paralogs were corrected using our custom correction probes (details are described in GPL18374 of Gene Expression Omnibus (GEO). The data sets of the microarray experiments were deposited at GEO (GSE68173).

### Scaling of mRNA numbers

Geometrical means of mRNA numbers for 5,157 protein-coding genes of *S. pombe* were calculated from four independent experiments. Scaling of mRNA numbers depended on the type of analysis as follows.

1. mRNA distribution analysis: we selected 4,678 genes in the 1 × N-EMM2 culture and 4,705 genes in the 1/100 × N-EMM2 culture that were reproducibly detected in the four independent experiments. The geometric mean was multiplied with a constant value so that the number of mRNA for non-RP genes totaled one million.
2. Simulation analysis: after performing (1), the scaled values of the paralogs of the same ribosomal subunit were summed for each of the 79 subunits.
3. Comparison between the 1 × N-EMM2 and 1/100 × N-EMM2 cultures: we selected 4,722 genes that were reproducibly detected in the four independent experiments either in the 1 × N-EMM2 culture or in the 1/100 × N-EMM2 culture. The geometric mean was scaled so that the total number of mRNA of the 4,722 genes totaled one million.

### Fitting analysis of mRNA distribution

Theoretical distributions were fitted to the empirical mRNA data by first removing RPs from the dataset to only account for the values contributing to the primary peak. An empirical cumulative distribution function (CDF) was generated from the normalized histogram and used as the basis for comparison with the following theoretical CDFs.

The *log-normal* (LN) distribution has two parameters 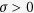 and 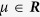, and its CDF is defined as:


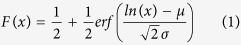


where *erf* is the Gaussian error function. The CDF of the Gamma distribution is defined as:


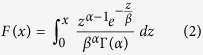


with the shape and rate parameters α, β > 0, respectively, and the gamma function Γ. For both, LN and Gamma, maximum likelihood estimators were used for deriving the distribution parameters.

We also compared the empirical data with two less well-known distributions that characterize the left and right tails with heavy tail parameters. The double Pareto (dP) distribution^14^ has the parameters α and β for both tails connected at X_0_. Its CDF is defined as:


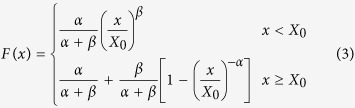


which we extended from its original definition^14^ to permit values other than X_0_ = 1. Finally, we also considered the double Pareto log-normal (dPLN) distribution^15^,^16^, which, as well as having heavy left and right tails with parameters α and β, also has a log-normal body, given by the log-normal mean ν and variance τ^2^.

Figure 1c,d show that while some fitted distributions matched either the left or the right tails rather well, only dPLN achieved an overall good fit for both tails. To validate the goodness-of-fit of each distribution with the original data, we performed a one-sided Kolmogorov–Smirnov (KS) test, which rejected all distributions as a match at 5% significance level except for dPLN with a p-value of 0.162. The estimated parameters obtained from the fitting were as follows: LN (μ = 4.16, σ = 1.29), Gamma (α = 0.51, β = 430.34), dP (α = 0.97, β = 0.83, X_0_ = 63.88), and dPLN (α = 1.01, β = 1.46, ν = 3.83, τ = 0.61).

## Additional Information

**How to cite this article**: Chikashige, Y. *et al.* Cellular economy in fission yeast cells continuously cultured with limited nitrogen resources. *Sci. Rep.* **5**, 15617; doi: 10.1038/srep15617 (2015).

## Supplementary Material

Supplementary Notes

## Figures and Tables

**Table 1:** Genes having more than double the scaled mRNA numbers in the 1/100 × N-EMM2 culture compared with the 1 × N-EMM2 culture.

GeneName	Mata *et al.*, 2002	Description	logratio-exp1	logratio-exp2	logratio-exp3	logratio-exp4
isp4	induced	OPT oligopeptide transporter family Isp4	2.91	3.28	3.65	3.09
SPAC1F8.04c	induced	hydrolase (predicted)	2.21	2.31	2.96	2.39
mmf2	induced	homologous Pmf1 factor 1, implicated in isoleucine biosynthesis (predicted)	2.02	2.04	2.96	2.30
isp6	induced	vacuolar serine protease Isp6	1.52	1.74	2.85	1.39
mei2	induced	RNA-binding protein involved in meiosis Mei2	1.59	1.98	1.78	1.84
SPAC869.04	induced	formamidase-like protein (predicted)	9.66	9.64	11.42	10.38
SPAC869.03c	induced	urea transporter (predicted)	7.41	7.52	9.19	7.97
SPBPB2B2.01	induced	amino acid permease (predicted)	4.47	4.80	5.96	5.04
SPAC869.01	induced	amidase (predicted)	3.72	3.06	6.03	4.57
SPCC285.05	induced	purine nucleoside transmembrane transporter (predicted)	2.46	2.46	3.34	2.72
SPBC800.11	induced	inosine-uridine preferring nucleoside hydrolase (predicted)	1.84	1.91	3.50	2.70
SPAC11D3.03c	induced	conserved protein	1.90	1.83	3.21	2.66
urg2	induced	uracil phosphoribosyltransferase (predicted)	1.73	2.00	3.56	2.59
SPBPB2B2.05	induced	peptidase family C26 protein	2.21	2.42	3.28	2.58
SPBC1683.12	induced	nicotinic acid plasma membrane transporter (predicted)	2.06	2.03	3.60	2.53
SPAC186.03	induced	L-asparaginase (predicted)	2.59	2.62	2.96	2.19
isp7	induced	2-OG-Fe(II) oxygenase superfamily protein	1.64	1.96	2.85	2.03
SPCC191.05c	induced	nucleoside 2-deoxyribosyltransferase (predicted)	1.33	1.47	2.74	1.98
SPBC1773.12	induced	transcription factor (predicted)	1.80	1.77	3.81	1.96
SPAC11D3.17	induced	zf-C2H2 type zinc finger protein	1.70	1.81	2.56	1.89
SPCC417.10	induced	dipeptide transmembrane transporter (predicted)	1.44	1.70	3.19	1.86
per1	induced	plasma membrane amino acid permease Per1	1.77	1.82	2.14	1.86
uga1	induced	4-aminobutyrate aminotransferase (GABA transaminase)	1.54	1.82	2.57	1.82
SPAC1039.08	induced	serine acetyltransferase (predicted)	1.46	1.53	2.81	1.82
SPAC323.07c	induced	MatE family transporter (predicted)	1.01	1.08	2.15	1.63
SPCC1223.09	induced	uricase (predicted)	1.25	1.63	2.54	1.60
SPBPB2B2.06c	induced	phosphoprotein phosphatase (predicted)	1.63	1.82	1.63	1.60
SPAC977.13c	induced	hydrolase, pseudogene	2.33	2.44	3.34	1.59
SPAC922.06	induced	3-oxoacyl-[acyl-carrier-protein]reductase (predicted)	1.19	1.19	2.45	1.58
SPAC1039.07c	induced	aminotransferase class-III, unknown specificity	1.10	1.33	2.84	1.58
SPBC1683.06c	induced	uridine ribohydrolase (predicted)	1.54	1.48	2.71	1.53
SPCC1494.01	induced	iron/ascorbate oxidoreductase family	1.07	1.32	2.06	1.51
SPCC550.07	induced	acetamidase (predicted)	1.06	1.03	2.25	1.39
SPCC1450.07c	induced	D-amino acid oxidase (predicted)	1.21	1.40	2.08	1.20
SPAC1399.01c	induced	membrane transporter (predicted)	1.15	1.39	2.10	1.07
SPCC417.12	middle	carboxylesterase-lipase family protein	1.18	1.33	2.05	1.40
mug180	middle	esterase/lipase (predicted)	1.74	1.72	2.21	1.23
urg1	late	GTP cyclohydrolase II (predicted)	2.90	3.17	5.31	4.12
isp5		amino acid permease Isp5	3.49	3.59	4.83	4.02
str3		siderophore-iron transporter Str3	3.36	3.65	3.62	2.95
SPBPB21E7.09		L-asparaginase (predicted)	1.95	2.02	3.03	2.67
put4		proline specific plasma membrane permease Put4 (predicted)	2.19	2.33	3.32	2.58
cta3		P-type ATPase, calcium transporting Cta3	3.86	4.43	4.71	2.56
SPBC13A2.04c		PTR family peptide transporter (predicted)	1.99	2.32	2.93	2.10
amt1		ammonium transporter Amt1	1.64	2.01	2.06	1.93
SPBC1683.02		adenine/adenosine deaminase family (predicted)	1.38	1.53	2.60	1.83
mug146		meiotically upregulated gene Mug46	1.26	1.29	1.77	1.69
arg7		argininosuccinate lyase	1.12	1.13	2.25	1.63
SPCC320.14		threo-3-hydroxyaspartate ammonia-lyase (predicted)	1.02	1.26	1.77	1.58
SPCC74.04		amino acid permease (predicted)	1.65	1.84	2.94	1.56
frp1		ferric-chelate reductase Frp1	1.91	1.96	1.82	1.51
SPAC11D3.18c		nicotinic acid plasma membrane transporter (predicted)	1.06	1.17	2.47	1.50
SPCC965.11c		amino acid transporter (predicted)	1.37	1.78	2.23	1.46
SPCC965.12		dipeptidyl peptidase (predicted)	1.51	1.56	2.02	1.34
SPBC1773.17c		glyoxylate reductase (predicted)	1.13	1.07	1.81	1.31
SPBC23G7.13c		urea transporter (predicted)	1.09	1.26	1.96	1.23
SPAC11D3.07c		transcription factor (predicted)	1.05	1.43	2.11	1.17
frp2		ferric-chelate reductase Frp2 (predicted)	1.44	1.44	1.78	1.16
SPAC13F5.07c		zf PARP type zinc finger protein	1.18	1.70	2.04	1.07

The labels “induced”, “middle”, and “late” stand for “starvation- or pheromone-induced”, “middle-meiosis”, and “late-meiosis”, respectively, as described previously^7^. The label “log-ratio” stands for the logarithm to base 2 of the ratio of the relative mRNA number of the 1/100 × N-EMM2-cultured cells over the 1 × N-EMM2-cultured cells. The 59 genes shown increased their relative mRNA numbers by more than twice in the 1/100 × N-EMM2 culture compared with the 1 × N-EMM2 culture; this result was reproduced in four independent experiments.
