# Supplementary material for: Cellular economy in fission yeast cells continuously cultured with limited nitrogen resources
Source: Sci Rep. 2015 Oct 21;5:15617. doi: 10.1038/srep15617 (PMC4614384; doi:10.1038/srep15617)
Supplement: Supplementary Notes [file srep15617-s1.pdf]

Supplementary Information

Cellular economy in fission yeast cells continuously cultured with limited nitrogen resources

Yuji Chikashige[1], Shin'ichi Arakawa[2], Kenji Leibnitz[3], Chihiro Tsutsumi[1], Chie Mori[1], Hiroko Osakada[1], Masayuki Murata[2], Tokuko Haraguchi[1] and Yasushi Hiraoka[4]*

[1]Advanced ICT Research Institute Kobe, National Institute of Information and Communications Technology, 588-2 Iwaoka, Iwaoka-cho, Nishi-ku, Kobe 651-2492, Japan.

[2]Graduate School of Information Science and Technology, Osaka University, 1-5 Yamadaoka, Suita, Osaka 565-0871, Japan.

[3]Center for Information and Neural Networks, National Institute of Information and Communications Technology, 1-4 Yamadaoka, Suita, Osaka 565-0871, Japan.

[4]Graduate School of Frontier Biosciences, Osaka University, 1-3 Yamadaoka, Suita, Osaka 565-0871, Japan.

***Correspondence:** Email: hiraoka@fbs.osaka-u.ac.jp; phone: +81 6 6879 4621; fax: +81 6 6879 4622

Supplementary Notes

Optimization of hybridization conditions in DNA microarray experiments for *Schizosaccharomyces pombe*

To obtain accurate measurements of the mRNA levels using the DNA microarray, we first optimized the concentrations of the hybridization targets used in our experiments. We used the 8 × 60K format DNA microarray produced by Agilent technology, and followed the manufacturer's experimental procedure except for the target concentration in the hybridization reactions. The target concentration for the 8 × 60K format array recommended by the manufacturer is 0.6 μg of labeled cRNA per 50 μL. We denoted this concentration as 1×, and used the target diluted to 1/8, 1/12, 1/16, and 1/20 (Fig. S1). We used an 8 × 60K format array that includes 60617 probes (excluding the manufacturer's control probes) designed for 5094 protein-coding genes and 1513 non-coding RNA genes of the fission yeast *S. pombe*. The x-axis of Fig. S1 represents the rank of signal values in the descending order (*gProcessedsignals* value defined by the manufacturer's software Feature Extraction) of the 1/16× diluted experiment. The y-axis represents the relative signal value of each probe in each concentration when the signal value of the 1/16 concentration was normalized to 1/16. The signal values of the top 20000 probes were plotted. Each signal value of probes below the 2000th was approximately proportional to the dilution rate. However, the signal values of the top 2000 probes in the 1/8× diluted experiment were lower than the values expected from the dilution rate. A similar tendency was found in the 1/12 dilution experiment for the top 1000 probes. On the other hand, the ratio of the signal value in 1/16 dilutions and 1/20 dilutions was approximately constant in all probes.

These results indicate that the probes for the high-expression genes were saturated to the target when the target dilution rate was low. Thus, we concluded that an approximate 1/16 dilution is necessary to avoid saturation in quantitative measurements using the Agilent DNA microarray for *S. pombe.*

Next, we compared our data from the 1/16 dilution experiment and the expression data for *S. pombe* measured under similar conditions registered in a public database. Fig. S2 shows the scatter plot of the mRNA levels in our 1/16 experiment and data from other platforms registered in the database (GSE52759 in the left panel and GSE19020 in the right panel) in double logarithmic scales, where each dot corresponds to one gene. In both cases, the signal value of the high-level expression region in the registered data was lower than the value of our experiment measured while avoiding the saturation of probes, as mentioned above. These results demonstrate that lowering the target concentration is necessary for the accurate measurement of high-expression genes. The improved quantitative accuracy in our experiments has made it possible to determine the profile of high-expression genes in this study.

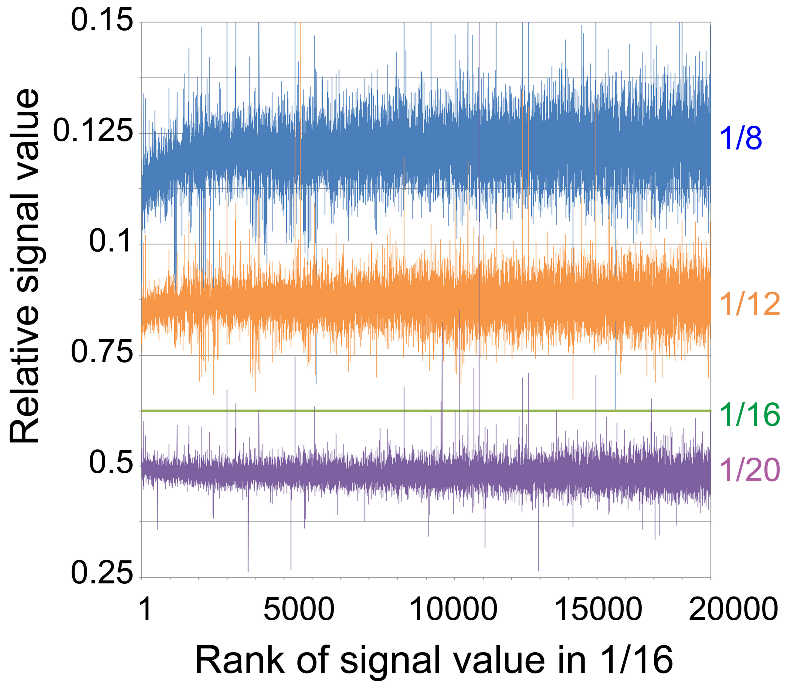

Figure S1. Saturation of probes for the high-expression genes with their targets.

Data used here are GSM1665061-4 in GSE68173.

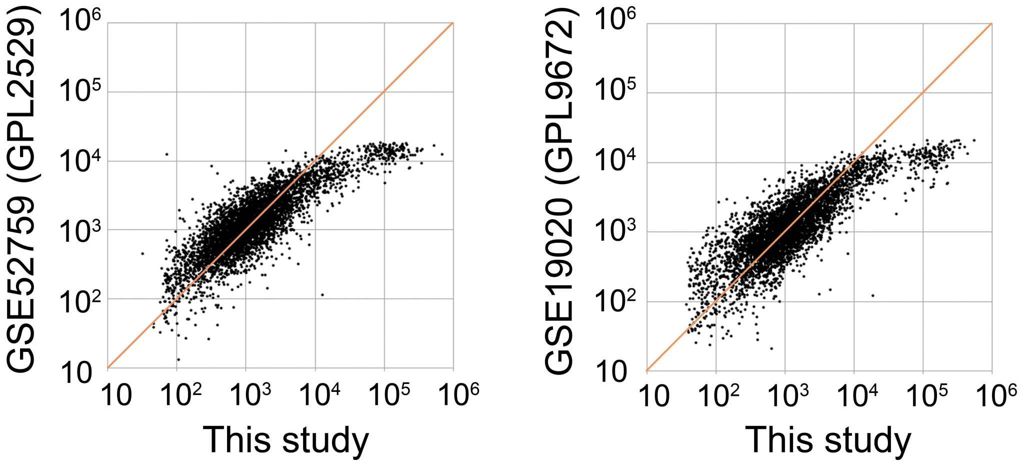

Figure S2. Comparison with data from other platforms registered in the database. Data used here for the y-axis are averages of three samples, GSM1275656-8 in GSE52759 (left, EMM2 at 30°C), and averages of four samples, GSM470527-8 and GSM470534-5 in GSE19020 (right, YE at 30°C); data for the y-axis are averages of three samples, GSM1665057-9 (left, EMM2 at 33°C) and GSM1665060 (right, YES at 30°C).
